# Aortoesophageal fistula following thoracic branch endoprosthesis for cryptogenic penetrating aortic ulcer in a patient on pembrolizumab

**DOI:** 10.1016/j.jvscit.2025.101837

**Published:** 2025-05-29

**Authors:** Sarah Kim, Patrick D. Conroy, Mark Zemela, Bruce L. Tjaden

**Affiliations:** a Cooper Medical School of Rowan University, Camden, NJ; b Department of Vascular and Endovascular Surgery, Cooper University Hospital, Camden, NJ

**Keywords:** Aortoesophageal fistula, Pembrolizumab, Penetrating aortic ulcer, Thoracic branch endoprosthesis

## Abstract

Penetrating aortic ulcers are uncommon and life-threatening. We describe the case of a 67-year-old man who presented with fever and chills and was found on computed tomographic scan to have a thoracic penetrating aortic ulcer. The patient underwent placement of a thoracic branch endoprosthesis, with a postoperative course complicated by ileocolic pseudoaneurysm, progressive aortitis, and a fatal aortoesophageal fistula. The management of penetrating aortic ulcer in the setting of multiple confounding factors is discussed in the context of this patient’s complex medical presentation, with a focus on the potential role of pembrolizumab in his disease progression.

A penetrating aortic ulcer (PAU) is a focal breach in the lumen wall of the aorta at the site of an atherosclerotic plaque. Luminal flow penetrates through the internal elastic lamina into the media. Patients with PAUs are typically older and have atherosclerotic disease as well as other vascular risk factors. Infectious cases may also occur, either in the setting of systemic bacteremia seeding a pre-existing aortic pathology, or a primary aortic infection leading to focal wall disruption. Large, irregular, progressive, infected, or symptomatic PAUs may be life threatening, and require urgent or emergent intervention. Here, we discuss the management of PAUs in a scenario filled with complicated considerations, with an emphasis on the potential role of pembrolizumab.

## Case report

We present the case of a 67-year-old male with a past medical history including hypertension, hyperlipidemia, multivessel coronary artery disease status post percutaneous coronary intervention 10 years prior (on Plavix), and stage IV non-small-cell lung cancer, being treated with carboplatin, pemetrexed, and pembrolizumab via a left chest wall port (last administered 2 days prior to admission).

**Fig 1:**
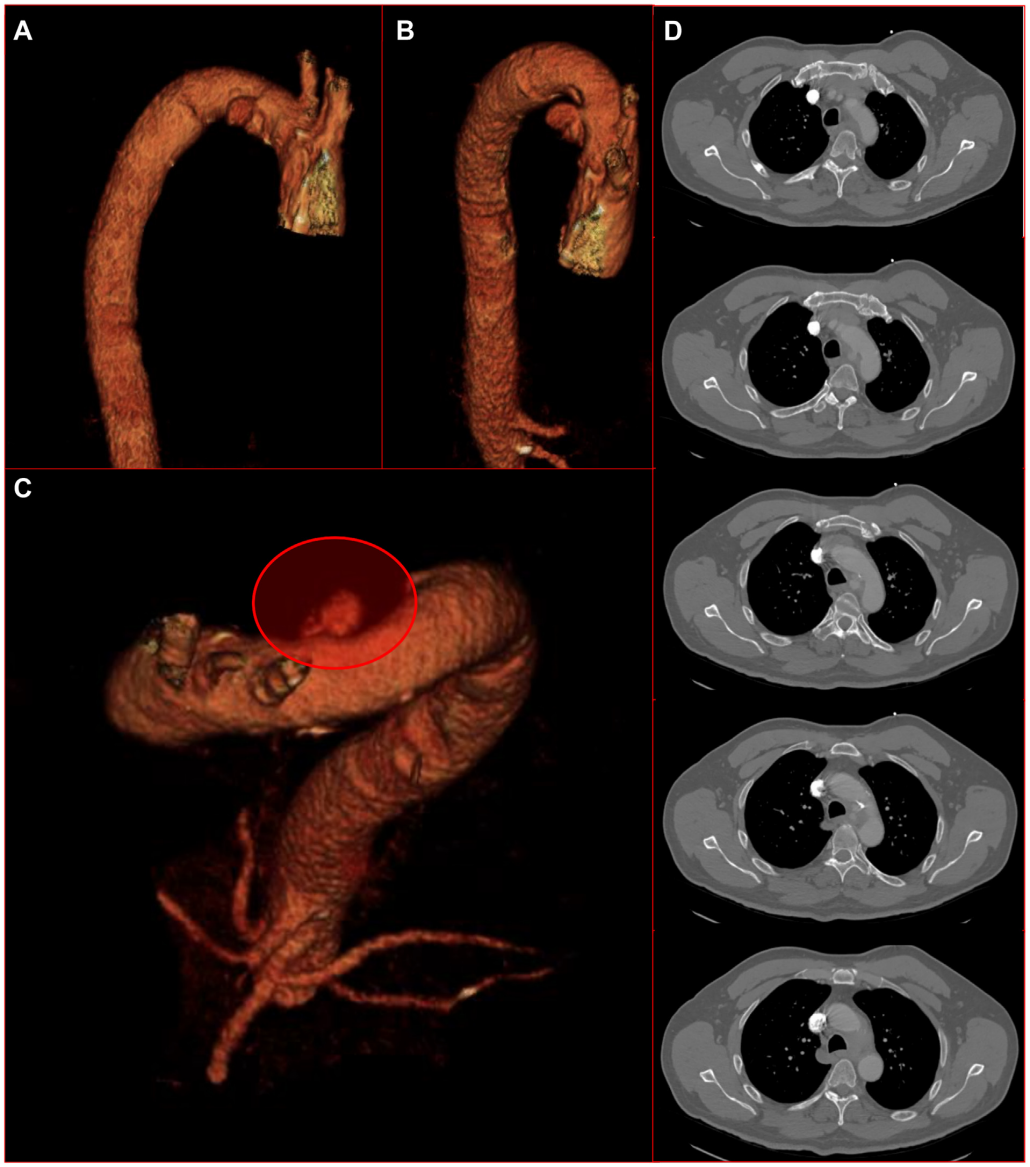
Index diagnostic computed tomography (CT) angiogram revealing posteromedial penetrating aortic ulcer (PAU) vs pseudoaneurysm. Three-dimensional reconstructions of the preoperative CT angiogram. **(A)** Posterior view (right anterior oblique [RAO], 100; cranial [CRA], 10). **(B)** Posterior view (RAO, 70; CRA 60). **(C)** Superior (left anterior oblique [LAO], 50; CRA, 70). **(D)** Axial CT angiography series.

He presented to an external hospital’s emergency department with fever and chills for 2 days. He was positive for systemic inflammatory response syndrome and bronchiolitis. Computed tomography (CT) angiography scan revealed a thoracic PAU distal to the left subclavian artery (Fig 1, *A-D*). The patient was transferred to our hospital for higher level of care. The primary concern was for either an infected PAU, a PAU resulting from peri-aortic tumor or lymph node erosion into the aorta in the setting of lung cancer, or a paraneoplastic vasculitis.

Given the acuity of the patient’s presentation and the delay in care that would result from waiting from further imaging like a positron emission tomography or a tagged white blood cell scan usually taking at least 2 to 3 days at our institution, the decision was made to forego further testing and proceed with treatment. We discussed with the patient that endovascular treatment of the PAU would reduce the risk of rupture in the near-term, but that if this were an infected PAU, thoracic endovascular aortic repair (TEVAR) would merely be a “temporizing” measure. He agreed to proceed.

**Fig 2:**
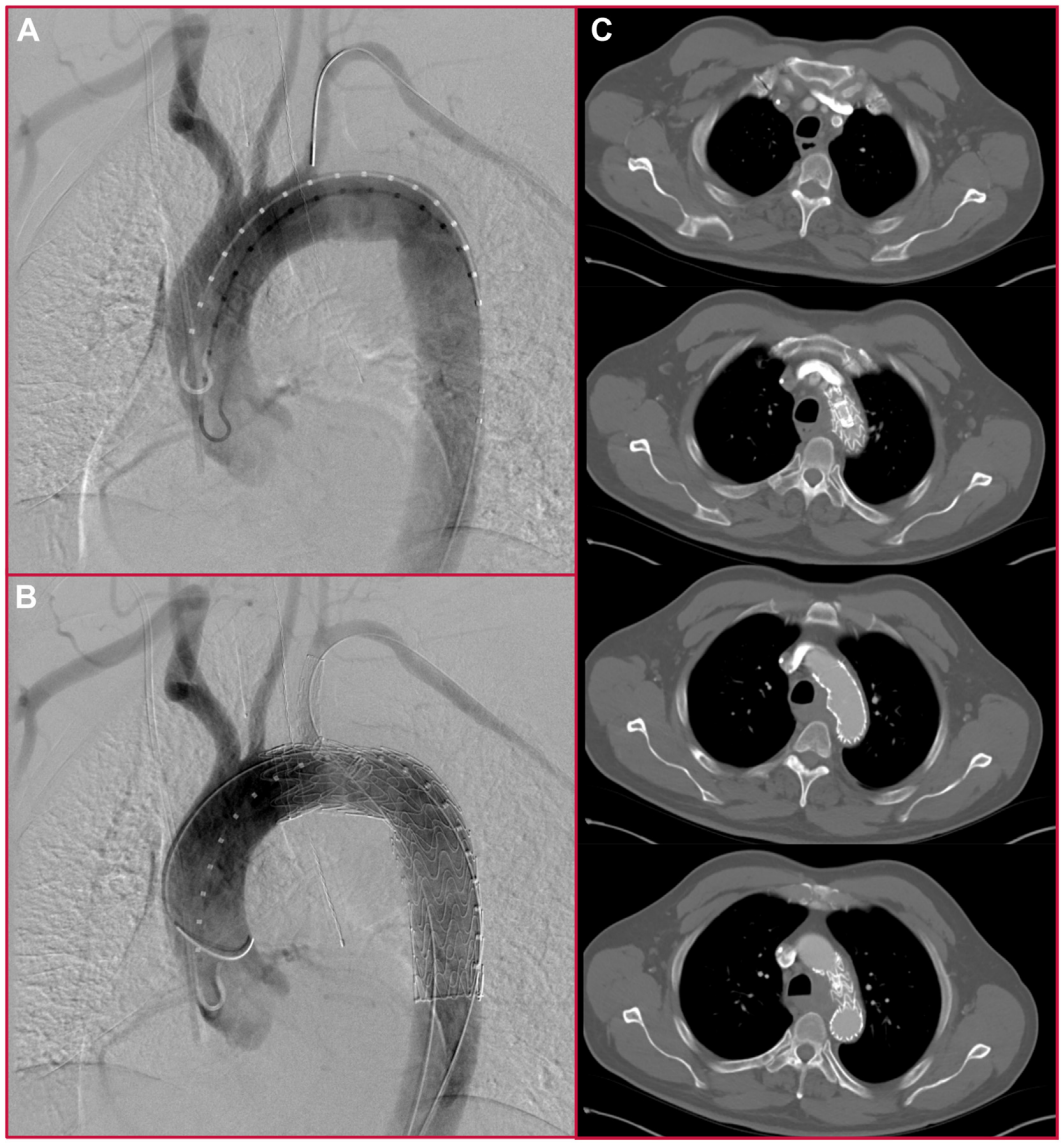
Intraoperative angiography. **(A)** Predeployment thoracic aortogram. **(B)** Completion thoracic aortogram. **(C)** Postoperative computed tomography (CT) angiogram revealing exclusion of posteromedial aortic pathology.

He underwent TEVAR using a branched thoracic device, covering zones 2 to 4 but preserving flow into the left subclavian artery via the thoracic branch (Fig 2, *A-C*). Blood cultures demonstrated no growth during his hospitalization or afterward. His postoperative course was uncomplicated, and he was discharged on postoperative day 3.

**Fig 3:**
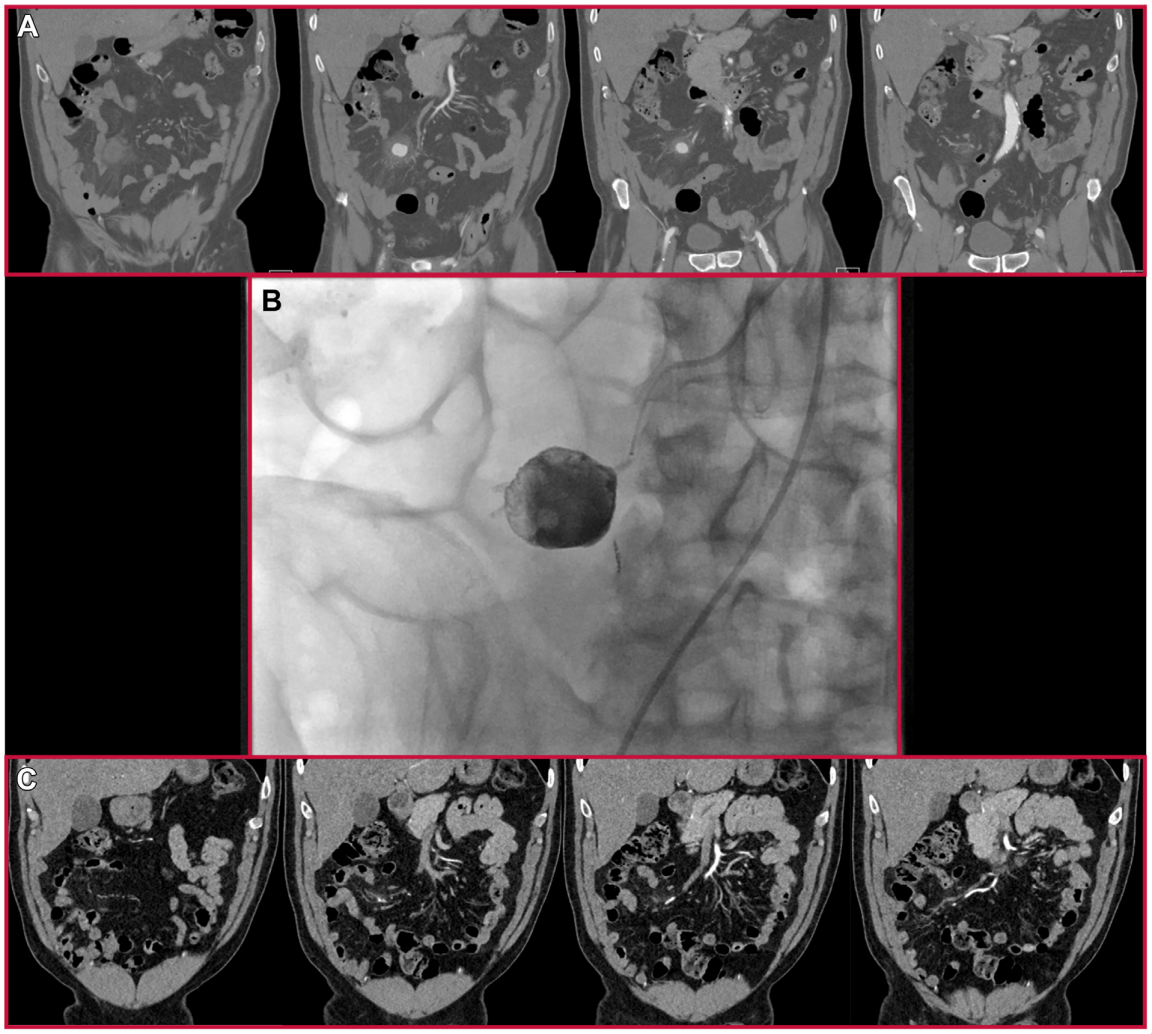
Ileocolic artery pseudoaneurysm that presented on readmission. **(A)** Diagnostic computed tomography (CT) angiogram revealing contained pseudoaneurysm. **(B)** Angiogram and coiling in the interventional radiology suite. **(C)** Review of index diagnostic CT angiogram revealing mesenteric inflammation.

Five days later (8 days from index operation), the patient presented to the emergency department with acute severe abdominal pain. CT scan demonstrated a 3-cm ileocolic branch pseudoaneurysm that was subsequently coil embolized by interventional radiology (Fig 3, *A-B*). On review of prior imaging, this pseudoaneurysm was not present on his initial CT scan, but some mesenteric inflammation in this area was noted (Fig 3, *C*). Blood cultures were again negative. He was discharged 3 days later with a presumed diagnosis of a diffuse paraneoplastic vasculitis vs a drug-induced vasculitis. No new anti-inflammatory medications were prescribed, but he was continued on his chemo-related oral dexamethasone, per Oncology’s recommendations. Outpatient follow-up with his oncologist was recommended shortly after discharge.

**Fig 4:**
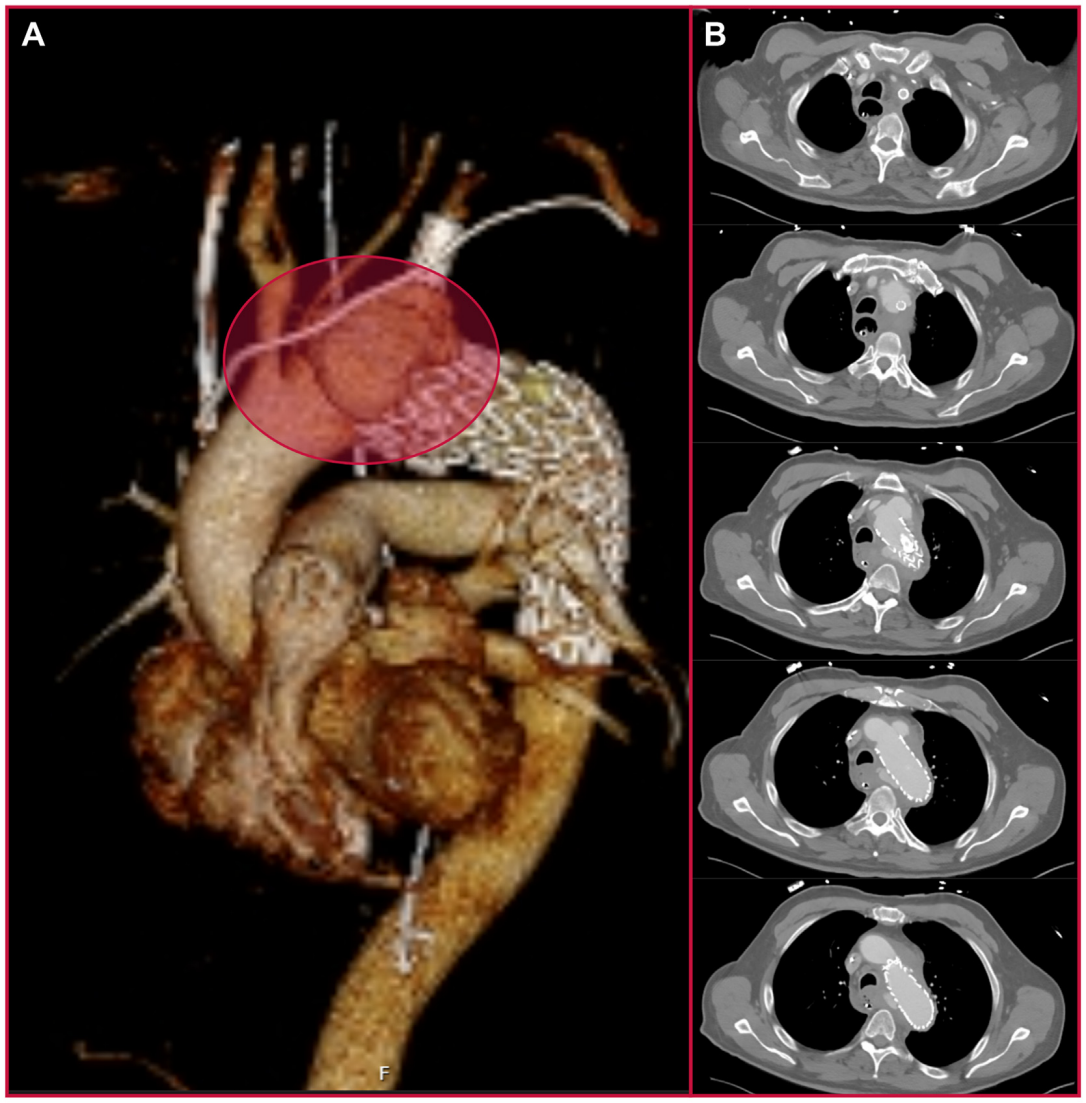
Final computed tomography (CT) scan revealing new anterolateral pseudoaneurysm with recurrent filling of original posteromedial aortic defect. **(A)** Three-dimentional reconstruction anterior view. **(B)** Axial CT angiography series.

Three weeks later (36 days from index operation), the patient presented with shortness of breath, chest tightness, chills, and malaise for 2 to 3 weeks. CT scan demonstrated development of edema in the upper mediastinum with thickening of the aortic wall, concerning for infectious aortitis with a new pseudoaneurysm at the level of the thoracic branch endoprosthesis (Fig 4, *A* and *B*). The patient was found to be bacteremic with fusobacterium. As no further endovascular or vascular surgical options were available, cardiothoracic surgery was consulted to evaluate for aortic explantation and homograft vs extra-anatomic reconstruction. He was deemed too high-risk for cardiothoracic surgical intervention and was recommended 6 weeks total of intravenous antibiotics with ceftriaxone.

Three weeks later (6 weeks from index operation), the patient presented to an external hospital with high volume hematemesis and was presumed to have developed an aortoesophageal fistula. The patient died shortly after being admitted to the external hospital’s emergency room. The patient’s wife consented to the publication of this anonymized case report.

## Discussion

This case illustrates the management of a PAU occurring in the setting of multiple confounding factors. This patient was actively undergoing chemotherapy to treat his stage IV non-small-cell lung cancer. His regimen included carboplatin, pemetrexed, and pembrolizumab. In addition to the usual concerns about immune suppression by chemotherapy, pembrolizumab specifically has been shown to have many other immune-mediated adverse effects, including aortitis and development of aortic aneurysm.^1^

By inhibiting programmed cell death protein 1, an immunosuppressive ligand, pembrolizumab causes enhanced T-cell activation against both tumor cells and normal cells, including the vascular endothelium. The first case report linking pembrolizumab to aortic pathology in 2022 by Ninomiya et al described a pembrolizumab-induced inflammatory ascending aortic aneurysm that was successfully surgically repaired.^1^ In the same year, a second case report by Chance et al reported pembrolizumab-induced aortitis of the transverse aortic arch that was successfully treated with high-dose prednisone and tocilizumab.^2^ Our case similarly demonstrates a temporal association of aortic pathology with pembrolizumab but differs in that our patient’s aortitis was treated with antibiotics, considering the primary concern for infectious etiology. Our patient also continued to develop an aortoesophageal fistula that he did not survive, illustrating the potentially fatal sequela of pembrolizumab-induced aortitis.

More recently, in 2024, Khan et al highlighted a case where pembrolizumab hastened aneurysmal degeneration and growth in a patient with a pre-existing ascending aortic aneurysm.^3^ In this case, the pembrolizumab may have contributed to the initial presentation of the patient, and/or may have contributed to subsequent aneurysmal degeneration after TEVAR. Other reports suggest that administering pembrolizumab to patients who are experiencing an infection may lead to even greater risk of developing an aneurysm.^4^ This is relevant given that our patient was positive for systemic inflammatory response syndrome and bronchiolitis on initial admission and had received pembrolizumab treatment 2 days prior.

In retrospect, it is unclear which was the “chicken” and which was the “egg” in this patient’s case. Did he have an underlying infection, worsened by chemotherapeutic immune suppression, leading to bacteremic seeding of an both an aortic PAU and a visceral pseudoaneurysm, which had formed due to pembrolizumab treatment? Did he have a systemic paraneoplastic or pembrolizumab-associated vasculitis leading to aortic and visceral artery degeneration, only later developing an infectious component related to immune suppression? Unfortunately, we could not elucidate the causal factor, and cannot definitively say what was the root of the issue.

Most importantly, this case serves as a humbling reminder of our limitations as surgeons. In cases of infected aortic pathology, or cases in which an unusual and otherwise untreatable condition is felt to have caused the aortic pathology (ie, cancerous invasion,^5^, ^6^, ^7^ Bacillus Calmette-Guérin aortitis after bladder cancer treatment^8^), endovascular therapy is often offered as a “temporizing” or “bridge” therapy.^9^^,^^10^ The assumption is generally that endovascular repair will solve the immediate problem, and “buy time” for appropriate infectious workup, chemotherapy or radiation treatment, etc. If nothing else, we often perform endovascular aortic repairs to prolong the patient’s meaningful time with their families. This is especially true in this patient’s case, given the already poor prognosis of his stage IV non-small-cell lung cancer diagnosis.

Moreover, the case presented here serves to illustrate that endovascular solutions are not panaceas. Our patient died of an aortoesophageal fistula a mere 5 weeks from initial diagnosis of his aortic pathology. His case illustrates the challenges of managing vascular complications particularly in oncology patients, and points to how future management must consider the timing of immune checkpoint inhibitors and endovascular procedures to prevent the catastrophic disease progression this patient experienced.

## Funding

None.

## Disclosures

None.
